# A Narrowband IoT Personal Sensor for Long-Term Heart Rate Monitoring and Atrial Fibrillation Detection

**DOI:** 10.3390/s24144432

**Published:** 2024-07-09

**Authors:** Eliana Cinotti, Jessica Centracchio, Salvatore Parlato, Emilio Andreozzi, Daniele Esposito, Vincenzo Muto, Paolo Bifulco, Michele Riccio

**Affiliations:** 1 Department of Electrical Engineering and Information Technologies, University of Naples Federico II, via Claudio, 21, 80125 Naples, Italy; eliana.cinotti@unina.it (E.C.); jessica.centracchio@unina.it (J.C.); salvatore.parlato@unina.it (S.P.); emilio.andreozzi@unina.it (E.A.); vincenzo.muto@unina.it (V.M.); paolo.bifulco@unina.it (P.B.); michele.riccio@unina.it (M.R.); 2 Department of Information and Electrical Engineering and Applied Mathematics, University of Salerno, via Giovanni Paolo II, 132, 84084 Fisciano, Italy

**Keywords:** medical IoT, atrial fibrillation recognition, narrowband IoT, cloud signal processing, personal ECG monitor

## Abstract

Long-term patient monitoring is required for detection of episodes of atrial fibrillation, one of the most widespread cardiac pathologies. Today, the most used non-invasive technique is Holter electrocardiographic (ECG) monitoring, which can often prove ineffective because of the short duration of recordings (e.g., one day). Other techniques such as photo-plethysmography are adopted by smartwatches for much longer duration monitoring, but this has the disadvantage of offering only intermittent measurements. This study proposes an Internet of Things (IoT) sensor that can provide a very long period of continuous monitoring. The sensor consists of an ECG-integrated Analog Front End (MAX30003), a microcontroller (STM32F401RE), and an IoT narrowband module (STEVAL-STMODLTE). The instantaneous heart rate is extracted from the ECG recording in real time. At intervals of two minutes, the sequence of inter-beat intervals is transmitted to an IoT cloud platform (ThingSpeak). Settled atrial fibrillation event recognition software runs on the cloud and generates alerts when it recognizes such arrhythmia. Performances of the proposed sensor were evaluated by generating analog ECG signals from a public dataset of ECG signals with atrial fibrillation episodes, the MIT-BIH Atrial Fibrillation Database, each recording lasting approximately 10 h. Software implementing the Lorentz algorithm, one of the best detectors of atrial fibrillation, was implemented on the cloud platform. The accuracy, sensitivity, and specificity in recognizing atrial fibrillation episodes of the proposed system was calculated by comparison with a cardiologist’s reference data. Across all patients, the proposed method achieved an accuracy of 0.88, a sensitivity 0.71, and a specificity 0.99. The results obtained suggest that the developed system can continuously record and transmit heart rhythms effectively and efficiently and, in addition, offers considerable performance in recognizing atrial fibrillation episodes in real time.

## 1. Introduction

Atrial fibrillation (AF) is one of the most widespread cardiac arrhythmias and it is mainly characterized by a change in heart rhythm (a complete list of all acronyms in the text can be found in Abbreviation Section). Clinical risk factors for AF include advanced age, diabetes, hypertension, congestive heart failure, rheumatic and non-rheumatic valve disease, and myocardial infarction [1]. AF is considered a 21st century cardiovascular disease epidemic [2]. In the literature, recent studies show that the incidence of AF has doubled globally in the last 30 years, partly attributed to the growing population of older individuals [3,4,5]. AF is currently acknowledged as the most prevalent cardiac arrhythmia and is associated with considerable complications and healthcare costs [6]. AF is most common in high-income countries such as the United States, Western Europe, and Australia. Unfortunately, a clear vision remains elusive due to the absence of community-based studies providing comprehensive information [7]. Early recognition of AF is of paramount importance for timely treatment [8]. This has promoted the development of various technologies for accurate personal monitoring of symptoms, the most important of which is an irregular heart rhythm.

In the medical field, new technologies are being studied to monitor and diagnose health-related disorders. Mobile health devices (mHealth) offer an opportunity for digital screening and, most importantly, enhance patient care [9]. Over the years, several mobile technologies have been developed to monitor heartbeats and detect arrhythmias. The analysis of electrocardiography (ECG) signals is the gold standard for the diagnosis of AF. With an ECG signal, the electrical activity of the heart can be analyzed and abnormal heart rhythms can be detected. AF ECG signals are characterized by many and inconsistent fibrillatory waves in place of normal P waves and an irregular heart rhythm [10]. A portable device used to record ECG signals is the Holter monitor. The Holter is a non-implantable device that allows ECG recordings for a limited time of about 24 to 48 h in non-clinical settings and provides valuable support in the diagnosis of AF [11]. Often, AF events may not appear during Holter recordings, resulting in failed early diagnosis.

To date, alternative devices for AF detection are available on the market which allow the users to perform intermittent monitoring when they feel an irregular heart rhythm. An example is the “Kardia mobile” (AliveCor, Mountain View, CA, USA) [12], which is a portable device that allows short-term ECG recordings from 5 min to 30 min. This device uses wireless communication with a personal device (smartphone or tablet). The Kardia mobile works properly up to 30 cm from the personal device; higher distances can lead to communication problems. The Kardia mobile requires contact with the subject’s skin, so it cannot be used in the case of some diseases, e.g., for Parkinson’s patients [13]. The device has been validated on patients with AF after an ablation procedure [14]. The main limitation of this kind of intermittent monitoring is that patients must voluntarily use the device when they feel arrhythmic heartbeats.

According to [15], long-term heart rate monitoring is better than intermittent heart rate monitoring. Current research is focused on the design of personal devices that can be used daily and continuously [16]. In new-generation monitors, loop recorders allow ECG tracing to be recorded for an extended period to diagnose hearth rhythm disorders. These devices offer a potential reduction of healthcare costs, because the possibility of remote monitoring improves the timing of diagnostics and consequently the follow-up strategy [17]. There are two different categories of loop recorders: implantable loop recorders (ILRs) and external loop recorders (ELRs) [18]. The ILRs are implantable and subcutaneous devices whose use requires surgery for both implantation and explantation of the device [17]. The use of these devices is more invasive for the patient, who must undergo both surgeries. The time duration of ECG monitoring with loop recorders depends on the type of device used. The expected monitoring duration with ILRs is approximately 28 months to a maximum of 3 years. This time interval is less for ELR devices, whose average lifetime is only a few weeks, and the total memory available to record the ECG trace is only a few minutes (minimum 6–10 min, maximum 30 h) [19].

One limitation of these devices is their memory, so recent studies have focused on developing devices that can connect to the cloud. Internet of Things (IoT) technology has been developing more in recent years. IoT refers to the network of physical objects—“things”—that are embedded with sensors, software, and other technologies for the purpose of connecting and exchanging data with other devices and systems over the Internet without human intervention. This makes it possible to connect different types of objects or sensors that can be used in daily life [20]. In recent years, the use of IoT objects has developed considerably; this is due to their features, such as low power consumption, long range, low cost, and security.

Using the IoT, a lot of information is shared, making it necessary to provide users with security regarding the shared data. It is essential to conduct formal verifications of IoT protocols. These verifications are categorized into various fields, as shown in the literature: functional checks, checks on security properties, suggestions for enhanced schemes including a priori security property checks, and implementation checks of protocols [21]. An example of formal verification techniques is shown in [22], even though it is very challenging for researchers to find a single method that can solve this problem. Other security protocols presented are PUF and POUF [23]. In the medical field, it is crucial to ensure data security. Since medical data are particularly sensitive, specific studies have been conducted on security protocols for healthcare products [24]. These studies have demonstrated that applying these protocols improves the security level of IoT devices. In the literature, several methods have been proposed to enhance the security protocols of narrowband Internet of Things (NB-IoT) devices [25,26].

In this study, the use of the NB-IoT is proposed. It is a narrowband radio technology specifically designed for the Internet of Things (IoT). With the rapid growth of the (IoT) market, low-power wide-area networks (LPWANs) have become a popular low-speed, long-range radio communication technology. Among them, the NB-IoT stands out, as it offers advantages in terms of latency and quality of service [27,28,29]. The NB-IoT is integrated into Long-Term Evolution (LTE) and it occupies a bandwidth of 180 kHz, corresponding to a resource block in LTE technologies [30]. To have wide-area coverage, NB-IoT technology must offer a high-quality signal which must have good performance, even in cases of crowded urban settings or in the presence of buildings. Therefore, the signal design aims to have a gain of ±20 dB compared with average cellular signals [31].

In healthcare, the use of the IoT is spreading rapidly, given the efficiency of the services it offers. Sensors connected by a wireless network enable them to assist patients by monitoring their health parameters continuously, and they offer remote observation and emergency procedures outside the hospital. These mobile devices and apps are now increasingly being used and integrated with telehealth and telemedicine through the Internet of Things in the medical field (mIoT) [32]. The NB-IoT is a simplified version of the IoT, and it can meet the tasks required by healthcare [33,34]. Doctors can have a wider view of the patient’s health condition using these smart sensors. NB-IoT sensors require very little power to operate, and battery life is estimated at about 13 to 15 years. The use of this technology and these devices would reduce healthcare costs, but an initial investment is needed to train doctors and technicians. As a negative aspect, the use of NB-IoT devices presents a limitation on the quality of images or information exchanged between the client and server; as an example, devices equipped with a bandwidth of 180 kHz cannot provide very high-quality performance. However, such shortcomings of the NB-IoT should be overcome in new versions. It is expected that the NB-IoT will be an integral part of the modern healthcare system [35]. To date, several devices using the NB-IoT have been developed in healthcare settings. For example, a patient fall monitoring system with immediate and simultaneous warning to four contacts has been designed [36], as it was a health monitoring platform based on the NB-IoT (NB-HIoT) [37].

In the literature, several studies have proposed wearable devices to detect atrial fibrillation, focusing on different aspects. They utilize various wearable devices that differ from the one presented in this work in terms of sensor type, signal analysis, cloud connectivity, and data analysis methods. The device described in [38] processes the ECG signal directly on board to detect AF events. In contrast, the device presented in this study does not perform any AF detection analysis at the sensor side; it solely receives inter-beat interval measurements from the MAX30003WING board and transmits them to a cloud service, where atrial fibrillation events are detected via an algorithm for the analysis of the inter-beat intervals series. By using LTE communication instead of Bluetooth, the proposed device eliminates the need for any additional nearby device (e.g., a smartphone or tablet). Other IoT devices typically utilize Bluetooth connectivity, as shown in [39]. Deep learning was also proposed for the detection of AF episodes, as an alternative to analytical algorithms [40]. The device described in [41] utilizes Bluetooth communication to transmit the entire ECG signal and not only the inter-beat intervals, thus requiring a much higher bitrate as compared to the prototype device described in this study. Another device leveraging Bluetooth is presented in [42] which employs machine learning for arrhythmia detection, specifically focusing on ventricular arrhythmias and not on atrial fibrillation. In addition, many studies use a photoplethysmography (PPG) signal instead of an ECG signal [43]. However, PPG sensors require much higher power consumption than ECG sensors. For example, PPG sensors used in smartwatches are only able to make occasional or discontinuous measurements, precisely because of the limitations imposed by power consumption. This can lead to missed detection of atrial fibrillation episodes. Instead, the prototype device described in this study allows continuous, long-term monitoring of inter-beat intervals, potentially overcoming the issue of missed AF detection. Other IoT devices described in the literature are based on Wi-Fi communication, as in [44], where a pulse sensor for AF detection is analyzed. This pulse sensor uses a bright red LED and a light detector to convert heartbeat fluctuations into electrical pulses. However, the use of Wi-Fi communication requires network coverage, which cannot always be guaranteed in all activities of the daily living of an individual. Moreover, in [44], none of the volunteers enrolled for the study had any AF episodes, so AF detection performance has not been assessed.

This study aims to design a non-implantable, wearable, integrated device to monitor heart rate with a focus on the early detection of AF episodes. The use of the NB-IoT is proposed, as it allows connection to the cloud for continuous heart rate signal processing without needing an additional device nearby.

## 2. Materials and Methods

**Figure 1:**
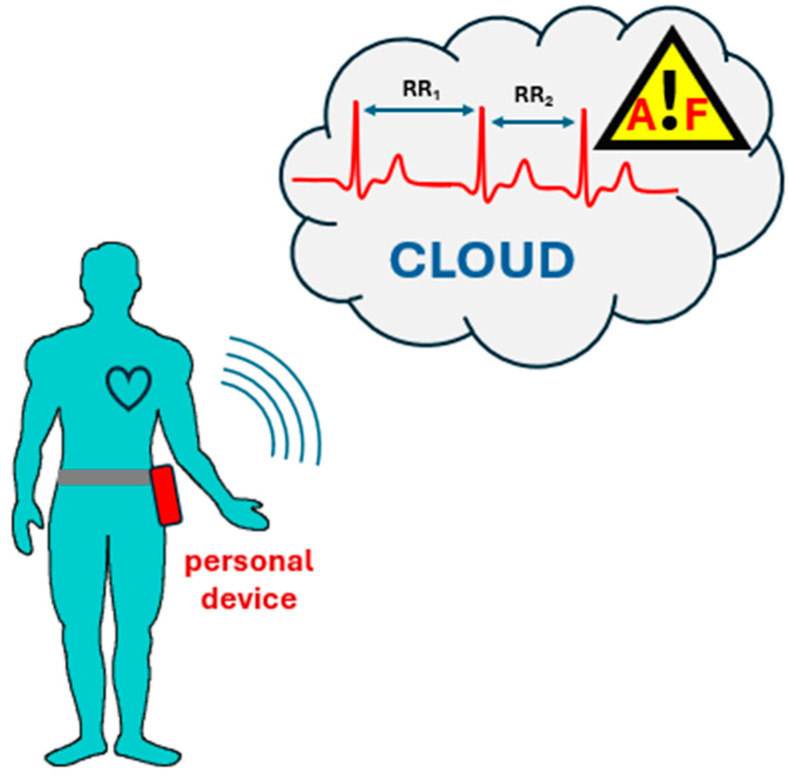
Architecture of the proposed system.

The developed system consists of a personal hardware device that acquires the ECG signal from the patient, pre-processes it, and wirelessly transmits it to a cloud platform, where a more complex algorithm is used to recognize AF episodes. Figure 1 schematically depicts the architecture of the system.

### 2.1. Personal Hardware Device

The developed prototype consists of 3 units: a data acquisition unit, a control unit, and a wireless Internet communication unit. The data acquisition unit (MAX30003WING, Analog Devices, Inc., Wilmington, MA, USA) is based on the MAX30003 chip, which includes a medical-grade ECG analog front-end and a digital pre-processing unit that processes the acquired ECG signal and provides inter-beat intervals estimates as output. The control unit (Nucleo-F401RE board, STMicroelectronics, Coppell, TX, USA) is based on the STM32F401RE microcontroller, which receives the inter-beat interval data from the data acquisition unit via the SPI protocol and transfers the data to the communication unit via the UART protocol. The communication unit consists of the STEVAL-STMODLTE (STMicroelectronics, Coppell, TX, USA) evaluation board for an LTE communication module, which provides Internet connectivity and allows data transfer to the cloud service. The personal hardware device continuously acquires the ECG signal from the subject but transmits, at set intervals, only the sequence of inter-beat time intervals. This requires a particularly low transmission rate (e.g., 2–4 bytes per second). The footprint of the entire prototype is 11 × 8 × 6 cm.

**Figure 2:**
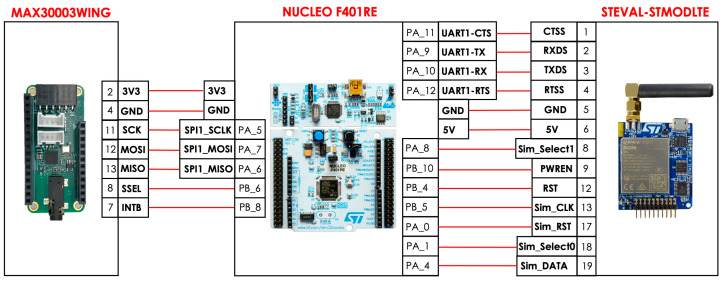
Hardware circuitry of the prototype architecture.

Table 1 shows the materials used for the prototype device. The electrical connections between the three boards are shown in Figure 2.

#### 2.1.1. The MAX30003 Board

The MAX30003 chip is a generic biopotential analog front-end specifically designed by Analog Devices for wearable applications [45]. It offers remarkable performance while ensuring reduced power consumption to extend battery life (85 μW at 1.1 V supply voltage). The MAX30003 provides a single biopotential channel and can be directly connected to skin electrodes. The evaluating board MAX30003WING was used. The biopotential amplifier offers high input impedance, DC coupling, and can handle large electrode voltage offsets: the electrodes can be directly connected to the board. This device has programmable gain, various filter options, and a high-resolution analog-to-digital converter. Furthermore, the chip offers an integrated, real-time implementation of the Pan-Tompkins QRS detection algorithm [46] on the acquired ECG signal.

When the personal device is turned on, the MAX30003 chip is programmed with the following settings. The gain of the analog front-end was set to 160 V/V, while the high-pass and low-pass digital filters were set to 0.5 and 40 Hz, respectively, in order to provide a standard monitor-grade ECG lead. The chip clock was set to 32 kHz; the sampling rate was set to 250 samples per second and the ADC resolution was 18 bits. The built-in Heart Rate Detection functionality was used to accurately find the R-peaks and to provide accurate instantaneous heart rate (i.e., inter-beat intervals). The main settings of the MAX30003 chip are summarized in Table 2.

#### 2.1.2. The NUCLEOF401RE Board

The Nucleo-F401RE is a development board designed by ST-Microelectronics [47]. It is an affordable and user-friendly development platform designed for rapid evaluation and prototyping. The board features a STM32F401RE microcontroller (32-bit microcontroller MCU) in a 64-pin LQFP64 package; it is an ARM Cortex M4 [48] processor operating at a clock frequency of 84 MHz. The device has 512 kB of flash memory and 96 kB of RAM, and two general-purpose direct memory access (DMA) controllers, each with 8 channels. The microcontroller board has the main task of communicating with both the ECG signal acquisition board and the wireless transmission board. The firmware for the control unit was developed in Mbed OS v.6.1. integrated development environment. On startup, the microcontroller starts the connection to the cellular network and establishes a connection with the cloud server (ThingSpeak) via the MQTT protocol (ver.2, QoS level 2). After the connection is established, the control unit configures the MAX30003 and starts the ECG acquisition. The microcontroller reads the inter-beat interval data provided by the MAX30003 via an interrupt, stores the data in a buffer, and sends a PUBLISH message to the cloud server with a payload consisting of the inter-beat intervals provided by the MAX30003.

#### 2.1.3. The STEVAL-STMODLTE Board

The STEVAL-STMODLTE [49] integrates an LTE cellular modem and a worldwide eSIM for continuous global coverage. The board features a penta-band right-angle stubby antenna (EU/US GSM/WCDMA), model W1900 from Pulse Larsen Antennas and the Quectel BG96 module, compatible with LTE Cat M1/Cat NB1/EGPRS technologies, achieving a maximum data rate of 375 kbps for downlink and uplink. This module supports global frequency bands and features extremely low power consumption. On the underside of the card is the socket for a micro-SIM card, given the possibility that the device offers to use both a virtual eSIM card and a physical card. The board was programmed to realize communication with the LPWAN radio protocol, as envisioned by the narrowband IoT technology.

### 2.2. Cloud Platform

The cloud platform is responsible for receiving inter-beat interval sequences and recognizing atrial fibrillation episodes by processing the data as they are received. The ThingSpeak IoT platform was adopted to implement the cloud service. Lorentz’s algorithm [50] implemented in MATLAB^®^ (The MathWorks, Inc., 1 Apple Hill Drive, Natick, MA, USA) is used to recognize AF episodes every 2 min.

#### ThingSpeak IoT Service

ThingSpeak is an IoT platform service with MATLAB^®^ Analytics [51] which allows real-time data streams to be collected, visualized, and analyzed in the cloud. With MATLAB^®^ Analytics integration, MATLAB code can be written and executed to perform preprocessing, visualization, and analysis. ThingSpeak supports HTTP or MQTT protocols.

### 2.3. Algorithms for Detecting AF

Several algorithms are available to detect AF episodes. AF detectors can be separated into two major classes: methods based on atrial activity analysis and methods based on ventricular response analysis. As an example, Mainardi et al. developed a method for automatic and real-time detection of AF [52]. Babaeizadeh et al. [53] presented another algorithm based on both ventricular and atrial response analysis. Lake and Moorman [54] presented an algorithm (i.e., CosEn) for the automatic detection of atrial fibrillation in a series of very short RR intervals, obtained from a single ECG lead. According to [55] the best methods for AF detection are: CosEn (Lake and Moorman) [54], MAD (Linker DT.) [56], and Lorenz (Sarkar et al.) [50]. Among them, the one that performs best on the MIT-BIH database [57] is Lorenz’s method, which always shows the best accuracy and the lowest number of false positives and false negatives. According to [58], Lorenz’s method is among the noteworthy methods that show good predictive value applicable in clinical settings. The Lorentz method was adopted in this study because of its effectiveness and accuracy in recognizing episodes of atrial fibrillation.

#### Lorenz Algorithm

**Figure 3:**
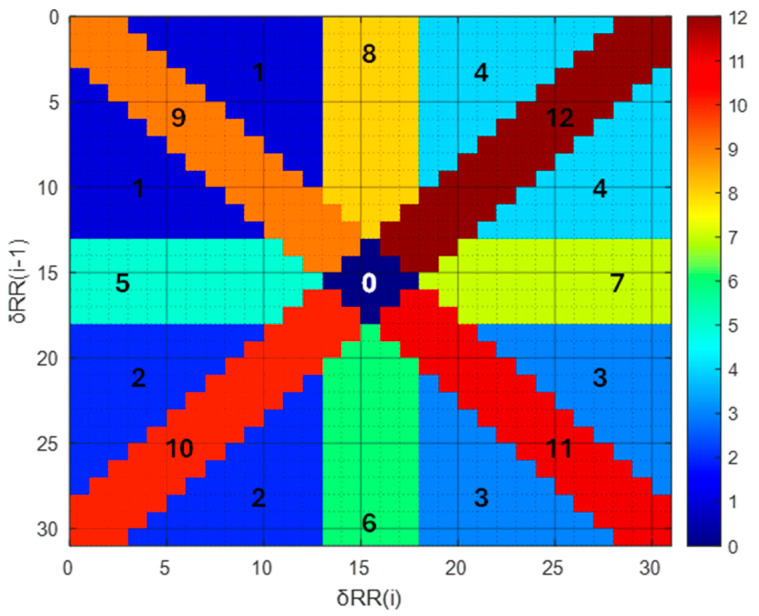
Partitioning of the Lorenz scatter plot domain in 13 regions.

The AF detection algorithm presented by Sarkar et al. [50] is based on the analysis of inter-beat interval (RR interval) time series via the Lorenz’s scatter plot. This plot is constructed from the time series of the differences in duration between successive couples of RR intervals. The difference in duration of a couple of RR intervals is defined as δRR(i) = RR(i) − RR(i − 1). The Lorenz’s scatter plot represents δRR(i) as the x-coordinate versus δRR(i − 1) as the y-coordinate. The scatter plot domain is limited to about −600 ms to +600 ms for both axes and it is quantized in 31 by 31 bins (see Figure 3). The algorithm proposed by Sarkar et al. [50] considers the RR intervals within an optimal time window of 2 min and calculates the occurrences of successive δRR in the scatter plot bins. Then, the occurrences are further evaluated based on 13 zones (or segments) of the scatter plot domain, which are numbered from 0 to 12 and colored with different hues in Figure 3. Region 0 is centered at the origin of the scatter plot and extends with a radius equal to 80 ms, which is related to the typical RR duration variability of Normal Sinus Rhythm (NSR).

The 2-min sequence of δRRs generates a bidimensional histogram of the occurrences of values associated with each bin. The algorithm to detect AF involves calculating the following indexes: *IrregularityEvidence* (1); *PACEvidence* (2); and *AFEvidence* (3).

*IrregularityEvidence* (1) assesses the distribution’s sparsity, observing a high value during AF and a low value during Normal Sinus Rhythm (NSR):

(1) $$IrregularityEvidence=\sum_{n=1}^{12}{BinCount}_{n},$$

where ${BinCount}_{n}$ counts the number of bins in region *n* of the 2-D histogram that are populated at least once.

*PACEvidence* (2) measures the evidence of compensatory pauses:

(2) $$PACEvidence=\sum_{n=1}^{4}\left({PointCount}_{n}-{BinCount}_{n}\right)+\;\sum_{n=5,6,10}{(PointCount}_{n}-{BinCount}_{n})-\sum_{n=7,8,12}{(PointCount}_{n}-{BinCount}_{n}),$$

where ${PointCount}_{n}$ counts the number of times bins are populated in region *n*.

(3) AFEvidence=IrregularityEvidence−OriginCount−2×PACEvidence,

where OriginCount is the count of the number of {δRR(i),δRR(i−1)} values in the bin containing the origin (numbered 0 in Figure 3).

By comparing the *AFEvidence* index with a predefined *AFThreshold* of 50, AF epi-sodes can be detected.

Lorenz’s algorithm can also provide recognition of other types of arrhythmias, such as atrial tachycardia (AT), series of premature atrial contractions, AT with regular ventricular response, AT with “group beating”, and AT with irregular ventricular response. The aim of this study was limited to detection of AF events only, but it is also possible to extend these analyses to different types of heart rhythm disorders.

### 2.4. AF ECG Dataset

**Figure 4:**
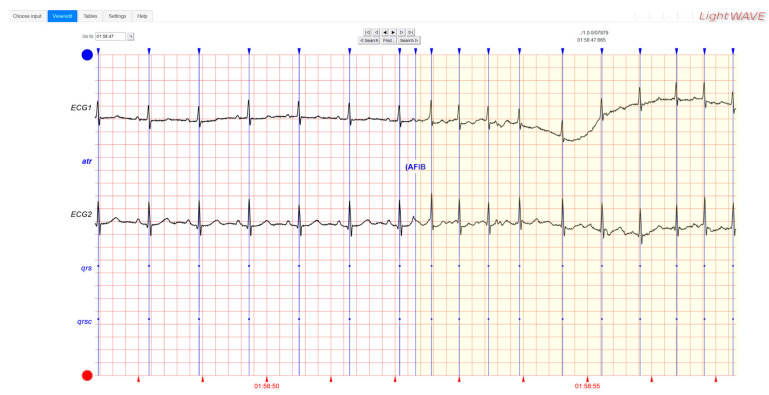
An example of ECG recordings from patient 07879. In the left part of the figure, the heart rhythm is normal. In the right part of the figure in yellow background, after the label “AFIB”, the heart rhythm is annotated as atrial fibrillating.

The performance of the developed system was evaluated by means of ECG signals containing episodes of atrial fibrillation accurately annotated by specialist cardiologists. The signals belonging to the publicly available “MIT-BIH Atrial fibrillation Database” [57] were considered. No other human subjects were involved in this study. The database contains ECG recordings of 23 patients with episodes of atrial fibrillation. The ECG signals were sampled at a rate of 250 samples per second with a 12-bit resolution across a voltage range of ±10 millivolts. The duration of each recording is approximately 10 h. For each recording, expert cardiologists recognized and annotated homogeneous ECG tracts corresponding to normal rhythms, episodes of atrial fibrillation, and other possible arrhythmias, marking exactly the start time and end time of each tract. This information constitutes the ground truth, i.e., the reference data, for the comparison tests performed. An example of ECG recordings is shown in Figure 4. Only the patient labelled 07859, exhibiting a particular type of atrial fibrillation with an unusual constancy of heart rhythm, was discarded. Therefore, the performance tests were carried out on 10-h ECG recordings of 22 AF patients.

### 2.5. Patient Simulator

**Figure 5:**
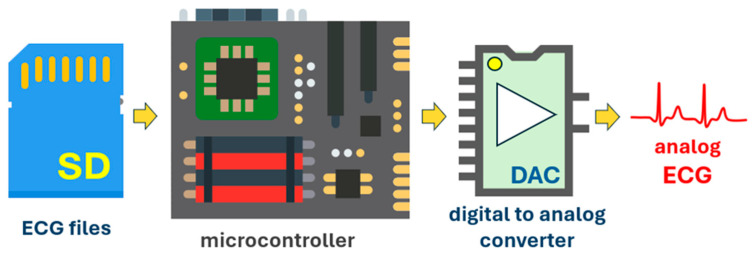
Sketch of the patient simulator circuitry.

An ad hoc circuit was made to reproduce ECG signals in analog form from the digital recordings stored in the MIT-BIH atrial fibrillation database. This was done to provide the input electrodes of the MAX30003 board with an analog ECG signal equivalent to that generated on the skin of a human subject. The system consists of an SD card (Gigastone, Irvine, CA, USA) reader, where the ECG database files have been stored, and a NUCLEO-F401RE board that reads the ECG data at the same frequency as the original recordings. The MCP4724 (Microchip Technology, Chandler, AZ, USA) is a 12-bit analog–digital converter that converts the data to analog signals. A low-pass filter with a cutoff frequency of 120 Hz follows the Digital to Analog Converter (DAC) to better reconstruct the analog signals. Finally, a voltage divider network lowers the voltage of these analogue signals to the level of potentials detectable on the human body. In Table 3, the materials of the patient simulator are shown. Figure 5 provides a schematic representation of the hardware made to simulate ECG signals as if they were generated by a real patient.

### 2.6. Statistical Analysis

The atrial fibrillation episode detections provided by the developed sensor were compared with the reference data (i.e., database annotations) in order to evaluate the overall performance of the system. Considering the 2-min sequences of inter-beat intervals, correctly recognized events (i.e., true positives, *TP*, and true negatives, *TN*) as well as false positives, *FP*, and false negatives, *FN*, were counted. Accuracy, sensitivity, and specificity of *AF* detection were computed for each patient recording, according to the following formulas:

(4) $$accuracy=\frac{TP+TN}{TP+TN+FP+FN}$$

(5) $$sensitivity=\frac{TP}{TP+FN}$$

(6) $$specificity=\frac{TN}{TN+FP}$$

These metrics are essential for assessing the capability of the proposed system to reliably detect atrial fibrillation episodes. Accuracy indicates the overall correctness of the detections, while sensitivity measures the proportion of true positive detections with respect to overall number of atrial fibrillation episodes. Specificity, on the other hand, measures the proportion of true negative detections with respect to the overall number of non-atrial fibrillation episodes. These metrics characterize the AF detection performance of the proposed system.

## 3. Results

The developed prototype system was able to perform all the functions for which it was designed. All tests took place at the University laboratory, and all intermediate steps were analyzed in detail. The hardware system was capable of correctly acquiring ECG signals, recognizing individual beats, calculating inter-beat intervals in real time, and transferring the few data obtained through sporadic connections to the cloud via the MQTT protocol. For example, if the patient has an average heart rate of 70 bpm, and each inter-beat interval is encoded in 2 bytes, the data packet to be transmitted every 2 min will amount to only 300 bytes. Obviously, the number of bytes may vary as the heart rate varies, but it will not vary significantly. However, the data are not transmitted continuously, but sporadically, so the transmitter is kept off most of the time, saving a lot on battery consumption. Tests were performed to evaluate the power consumption of the personal device. The average current draw was found to be about 10 mA. This absorption is mainly due to the use of the microcontroller and also depends on the fact that the firmware has not yet been optimized for power consumption. The recording duration obviously depends on the type of batteries used. For example, a small 2500 mAh battery provides about 10 days of operation.

**Figure 6:**
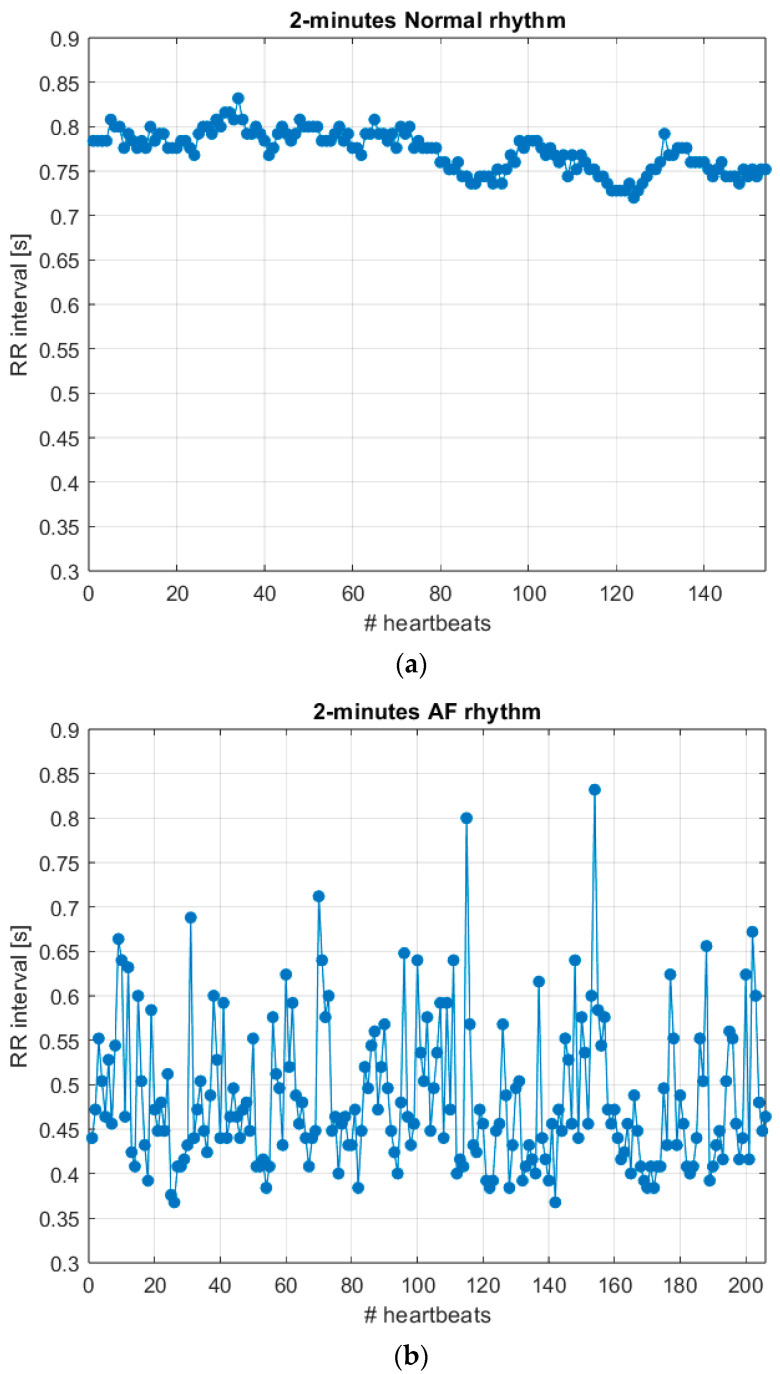
Examples of 2-min tachograms from patient 07879 in cases of (**a**) normal rhythm and (**b**) atrial fibrillation.

Data processing performed in the cloud classified inter-beat interval sequences of 2-min segments as normal or AF episodes. Figure 6 shows two examples of 2-min series of inter-beat intervals (i.e., tachograms) from patient 07879 in cases of normal rhythm and atrial fibrillation. The number of correct and incorrect classifications provided by the prototype device were determined by comparison with database annotations, considered as the ground truth. Then, the accuracy, sensitivity, and specificity of the AF detection were calculated for each patient recording. Table 4 shows the results obtained for each 10-h patient recording.

The accuracy, sensitivity, and specificity achieved across all patients were 0.878, 0.708, and 0.993, respectively. The results obtained are encouraging. Finally, Table 5 provides a comparison between the IoT system proposed in this study and other state-of-the-art systems described in previous studies, in terms of sensors, type of connection to the cloud, and AF detection algorithm.

## 4. Discussion

An innovative personal system capable of early detection of AF events was presented. The system consists of a hardware device that continuously acquires an ECG signal from a subject, computes inter-beat intervals, and periodically transmits the data to a cloud platform, where they are processed to recognize AF episodes. A proven heart rhythm analysis algorithm was implemented in the cloud for AF detection. The prototype hardware device made is of a small size and has low power consumption. The performance of the proposed system was assessed by using a patient simulator that reproduced real ECG signals from a public database of AF patients, supplied with annotations by expert cardiologists. The results obtained demonstrated the effectiveness of the proposed system and promising performance in recognizing AF episodes. Since the AF detection algorithm resides on the cloud, it can be easily upgraded without making changes to patients’ monitoring devices. There are not many studies in the literature addressing IoT systems for AF detection. Table 5 summarizes the main features of the few systems proposed in the literature. ECG-based systems have been tested with ECG data from public databases without providing any evidence about the performance of the ECG analog front-end. Instead, this study included ECG hardware testing by means of an ad hoc patient simulator. The solutions described in [38,39] have been tested only on four to five subjects from MIT databases, while this study analyzed the ECG data of 22 patients from the MIT-BIH database [57]. In [40], a deep learning algorithm for AF detection is proposed for IoT applications, but no hardware for ECG acquisition and transmission is described. The system described in [41] involves the transmission of the whole ECG signal, instead of the inter-beat intervals only, thus requiring a much higher bitrate than the prototype proposed in this study. The IoT system proposed in [42] has not addressed AF detection, but rather focused on other arrhythmias. The solution described in [43] has been tested only on a proprietary, non-public database, which prevents performance comparisons for AF detection. In [44], the authors described a system for bradycardia and AF detection, but none of the enrolled volunteers had any AF episodes, so the AF detection performance has not been assessed. Moreover, both solutions described in [43,44] are based on PPG sensors, which usually cause considerable power consumption, which is not compatible with continuous, long-term monitoring.

This study has some limitations. The prototype device was tested only on ECG data reproduced by the patient simulator, and not on actual patients. The transmitting device was tested only in a laboratory environment, and not during trips; hence, potential issues due to network coverage could not be assessed. The AF detection performance was tested on ECG data from the MIT-BIH Atrial Fibrillation Database [57] which were not heavily corrupted by noise. Moreover, the ECG data analyzed in this study comprised only AF episodes, so the specificity of AF detection was not assessed in the presence of additional cardiac arrhythmias. In a real-world scenario, motion artifacts, electromagnetic interferences, and muscle electrical activity may significantly degrade ECG signal quality and hinder the performance of the MAX30003 in heartbeat detection and inter-beat interval estimation.

A SWOT analysis was conducted to highlight the strengths, weaknesses, opportunities, and threats of the proposed monitoring system, and is reported in Table 6.

Future trends will certainly include the development of new device features. Once a rhythm abnormality is recognized, the morphology of the ECG tracing needs to be further analyzed to confirm the absence of coordinated atrial activity represented by P waves and/or to exclude recording artifacts. At present, the ECG signal is erased from time to time, retaining only the rhythm data. In the future, at each AF detection, the corresponding ECG trace will be automatically stored and transmitted to the cloud, so as to provide physicians with incontrovertible evidence of the discovery of transient AF episodes. In addition, it is planned to activate a messaging service aimed at both the patient and the physician to convey information to stakeholders in a timely manner and to trigger the acquisition of further information about the circumstances surrounding recognized episodes of AF. Once recognition of the occurrence of AF is made, the device could also be used in the future to monitor the effects of the therapy administered to the patient. The Lorentz algorithm is not limited to recognizing only atrial fibrillation but also different types of arrhythmias, such as atrial tachycardia with regular ventricular response, atrial tachycardia with “group beating”, atrial tachycardia with irregular ventricular response, premature ventricular contractions, etc. Upgrades of the SW code can provide additional information about other arrhythmic events to physicians and patients. Obviously, inter-beat interval processing can be improved by replacing the Lorentz algorithm with more sophisticated recognition techniques, such as machine learning and deep learning.

When utilizing IoT sensors for medical purposes, several critical regulatory and ethical considerations must be considered. These include compliance with medical device regulations, such as FDA guidelines in the U.S. and CE Marking requirements in the EU, depending on their intended use [59]. Additionally, adherence to data privacy laws, such as GDPR in the EU or HIPAA in the U.S., is essential when collecting and transmitting patient data via IoT sensors. Proper consent and secure data handling practices are of paramount importance. Future developments of the proposed system will address data security issues for clinical applications. Moreover, ensuring interoperability with existing healthcare systems and standards, such as HL7, is crucial for seamless integration and operation. Ethical considerations in using IoT sensors for healthcare include informed consent, data ownership and control, fairness in data processing, security and privacy safeguards, beneficence and non-maleficence in patient care, and ensuring equity in access to IoT-enabled healthcare services [60]. These principles aim to uphold patient autonomy, protect sensitive health data, mitigate biases, and promote fair and effective healthcare delivery.

Incorporating additional sensors to gather more data with a single hardware device is feasible and should be a key objective for future efforts. As an example, inertial measurement units could be included in the prototype device to estimate the type and level of physical activity of the subject. Moreover, accelerometers [61,62], gyroscopes [62,63], force sensors [64,65,66,67], and acoustic sensors [68] can be integrated in the proposed device to allow the concurrent acquisition of cardio-mechanical signals, thus enabling a more comprehensive evaluation of electrical and mechanical cardiac activity. Indeed, techniques like Seismocardiography, Gyrocardiography, Forcecardiography, and Phonocardiography can provide valuable additional information about cardiac diseases. Recently, substantial equivalence of these techniques to ECG data in the estimation of heart rhythms has been demonstrated by using template-matching algorithms for heartbeat detection. Therefore, these techniques can also be used to replace ECG data for heart rate monitoring and subsequent AF detection, with the advantage of not using electrodes connected to the patient’s skin.

## Figures and Tables

**Table 1:** List of materials used for the prototype.

Model	Manufacturer	Board Size
Max30003Wing	Analog Devices	50.8 × 23.6 mm
NucleoF401RE	STMicroelectronics	70.0 × 82.50 mm
STEVAL-STMODLTE	STMicroelectronics	78.0 × 40.0 mm

**Table 2:** Parameters’ settings.

**MAX30003 Settings**	
FMSTR clocks	32 kHz
Current magnitude	10 nA
**ECG Settings**	
Digital LPF cutoff	40 Hz
Digital HPF cutoff	0.5 Hz
ECG gain	160 V/V
Sample Rate	250 sps
**R-to-R Settings**	
WNDW	96 ms

**Table 3:** List of materials used for patient simulator.

Model	Manufacturer	Board Size
MCP4724	Microchip Technology	15.0 × 15.0 mm
NucleoF401RE	STMicroelectronics	70.0 × 82.50 mm
SD card	Gigastone	15.0 × 11.0 mm

**Table 4:** Accuracy, sensitivity, and specificity calculated for each patient.

Patient	Accuracy	Sensitivity	Specificity
04015	0.970	0.333	0.977
04043	0.816	0.141	1.00
04048	0.993	0.00	1.00
04126	0.951	0.00	0.993
04746	0.967	0.939	1.00
04908	0.935	0.269	0.996
04936	0.766	0.692	1.00
05091	1.00	//	1.00
05121	0.836	0.763	0.980
05261	1.00	1.00	1.00
06426	0.951	0.953	0.875
06453	0.993	0.500	0.996
06995	0.911	0.853	0.963
07162	1.00	1.00	//
07879	0.669	0.465	1.00
07910	0.997	0.979	1.00
08215	0.997	0.996	1.00
08219	0.823	0.333	0.995
08378	0.902	0.578	0.988
08405	0.905	0.868	1.00
08434	0.957	0.308	0.986
08455	0.832	0.754	1.00

**Table 5:** Comparison between the proposed method and existing similar state-of-the-art methods.

Study	Type of Connection	Sensor	Signal Analyzed	AF Detection Platform	AF Detection Method	Evaluation Metrics
Present study	LTE	ECG sensor	Inter-beat intervals	Cloud	Lorenz algorithm	Accuracy, sensitivity, specificity
[38]	Alarm notification to remote server	ECG sensor	ECG signal	On board	Algorithm based on RR irregularity and P-wave absence from ECG data	Sensitivity, specificity
[39]	Bluetooth	ECG sensor (AD8232)	ECG signal	Cloud	Deep learning algorithm	Accuracy, sensitivity, specificity
[40]	No	No	Heart rate signal	No	Deep learning algorithm	Accuracy
[41]	Bluetooth connection to smartphone	ECG patch	ECG signal	Cloud	Machine learning algorithm	Accuracy, sensitivity, specificity
[42]	Bluetooth connection to smartphone	Polar H10 ECG sensor	ECG signal	Cloud	No AF detection; ventricular arrhythmia detection via machine learning	Accuracy
[43]	No	PPG sensor	PPG signal	On board	Analytical algorithm to detect AF burden	Accuracy
[44]	Wi-Fi	Pulse sensor	Heart rate signal	Cloud	Algorithm developed by authors	–

**Table 6:** SWOT analysis.

	SUPPORT	OPPOSE
	**Strengths**	**Weaknesses**
**INTERNAL**	It is a low-cost solution for early detection of atrial fibrillation episodes Patient monitoring can be carried out for several hours/days No need for additional devices for data transfer (e.g., smartphone, tablets) Wider network coverage with respect to devices based on Wi-Fi Very low bitrate Flexibility in upgrading the software for AF detection on cloud server	AF detection only based on analysis of inter-beat intervals series, and not of ECG morphology Currently, the prototype cannot deal with lacks of network connection
	**Opportunities**	**Threats**
**EXTERNAL**	Memorizing ECG signal morphologies related to detected atrial fibrillation events Integrating additional sensors to gather more information about the patient Optimizing hardware and software to reduce size and power consumption	Significant motion artifacts or noises can hinder MAX30003 performance in heartbeat detection Potential absence of LTE network connection

## Data Availability

The original data presented in the study are openly available in [PhysioNet] at https://physionet.org/content/afdb/1.0.0/ (accessed on 24 June 2024).

## Abbreviation

**Acronym**	**Full Form**
**AF**	Atrial Fibrillation
**AT**	Atrial Tachycardia
**DAC**	Digital to Analog Converter
**DMA**	Direct Memory Access
**ECG**	Electrocardiography
**ELRs**	External Loop Recorders
**FN**	False Negative
**FP**	False Positive
**ILRs**	Implantable Loop Recorders
**IoT**	Internet of Things
**LPWAN**	Low-Power Wide-Area Network
**LTE**	Long-Term Evolution
**mHealth**	mobile Health devices
**mIoT**	Internet of Things in the medical field
**NB-HIoT**	Health monitoring based on NB-IoT
**NB-IoT**	Narrowband Internet of Things
**NSR**	Normal Sinus Rhythm
**POUF**	Protocols, Operations, Usage, and Formats
**PPG**	Photoplethysmography
**PUF**	Physical Unclonable Function
**SW**	Software
**TN**	True Negative
**TP**	True Positive

## References

[1] Benjamin E.J., Wolf P.A., D’Agostino R.B., Silbershatz H., Kannel W.B., Levy D. (1998). Impact of Atrial Fibrillation on the Risk of Death: The Framingham Heart Study. Circulation.

[2] Kornej J., Börschel C.S., Benjamin E.J., Schnabel R.B. (2020). Epidemiology of Atrial Fibrillation in the 21st Century: Novel Methods and New Insights. Circ. Res..

[3] Wu J., Nadarajah R., Nakao Y.M., Nakao K., Wilkinson C., Mamas M.A., Camm A.J., Gale C.P. (2022). Temporal Trends and Patterns in Atrial Fibrillation Incidence: A Population-Based Study of 3·4 Million Individuals. Lancet Reg. Health Eur..

[4] Schnabel R.B., Yin X., Gona P., Larson M.G., Beiser A.S., McManus D.D., Newton-Cheh C., Lubitz S.A., Magnani J.W., Ellinor P.T. (2015). 50 Year Trends in Atrial Fibrillation Prevalence, Incidence, Risk Factors, and Mortality in the Framingham Heart Study: A Cohort Study. Lancet.

[5] Vinciguerra M., Dobrev D., Nattel S. (2024). Atrial Fibrillation: Pathophysiology, Genetic and Epigenetic Mechanisms. Lancet Reg. Health Eur..

[6] Dilaveris P.E., Kennedy H.L. (2017). Silent Atrial Fibrillation: Epidemiology, Diagnosis, and Clinical Impact. Clin. Cardiol..

[7] Kornej J., Benjamin E.J., Magnani J.W. (2021). Atrial Fibrillation: Global Burdens and Global Opportunities. Heart.

[8] Patten M., Pecha S., Aydin A. (2018). Atrial Fibrillation in Hypertrophic Cardiomyopathy: Diagnosis and Considerations for Management. J. Atr. Fibrillation.

[9] Linz D., Gawalko M., Betz K., Hendriks J.M., Lip G.Y.H., Vinter N., Guo Y., Johnsen S. (2024). Atrial Fibrillation: Epidemiology, Screening and Digital Health. Lancet Reg. Health–Eur..

[10] Hagiwara Y., Fujita H., Oh S.L., Tan J.H., Tan R.S., Ciaccio E.J., Acharya U.R. (2018). Computer-Aided Diagnosis of Atrial Fibrillation Based on ECG Signals: A Review. Inf. Sci..

[11] Mubarik A., Iqbal A.M. (2024). Holter Monitor. StatPearls.

[12] Kardia Mobile 6L. https://alivecor.com/products/kardiamobile6l.

[13] Krzowski B., Skoczylas K., Osak G., Żurawska N., Peller M., Kołtowski Ł., Zych A., Główczyńska R., Lodziński P., Grabowski M. (2021). Kardia Mobile and ISTEL HR Applicability in Clinical Practice: A Comparison of Kardia Mobile, ISTEL HR, and Standard 12-Lead Electrocardiogram Records in 98 Consecutive Patients of a Tertiary Cardiovascular Care Centre. Eur. Heart J. Digit. Health.

[14] Tarakji K.G., Wazni O.M., Callahan T., Kanj M., Hakim A.H., Wolski K., Wilkoff B.L., Saliba W., Lindsay B.D. (2015). Using a Novel Wireless System for Monitoring Patients after the Atrial Fibrillation Ablation Procedure: The iTransmit Study. Heart Rhythm..

[15] Lee R., Mittal S. (2018). Utility and Limitations of Long-Term Monitoring of Atrial Fibrillation Using an Implantable Loop Recorder. Heart Rhythm..

[16] Kulkarni M.B., Rajagopal S., Prieto-Simón B., Pogue B.W. (2024). Recent Advances in Smart Wearable Sensors for Continuous Human Health Monitoring. Talanta.

[17] Bisignani A., De Bonis S., Mancuso L., Ceravolo G., Bisignani G. (2018). Implantable Loop Recorder in Clinical Practice. J. Arrhythm..

[18] Galli A., Ambrosini F., Lombardi F. (2016). Holter Monitoring and Loop Recorders: From Research to Clinical Practice. Arrhythm. Electrophysiol. Rev..

[19] Brignole M., Vardas P., Hoffman E., Huikuri H., Moya A., Ricci R., Sulke N., Wieling W., Auricchio A., Lip G.Y.H. (2009). Indications for the Use of Diagnostic Implantable and External ECG Loop Recorders. EP Eur..

[20] Li S., Xu L.D., Zhao S. (2015). The Internet of Things: A Survey. Inf. Syst. Front..

[21] Hofer-Schmitz K., Stojanović B. (2020). Towards Formal Verification of IoT Protocols: A Review. Comput. Netw..

[22] Krichen M. (2023). A Survey on Formal Verification and Validation Techniques for Internet of Things. Appl. Sci..

[23] Xu T., Wendt J.B., Potkonjak M. Security of IoT Systems: Design Challenges and Opportunities. Proceedings of the 2014 IEEE/ACM International Conference on Computer-Aided Design (ICCAD).

[24] Fazeldehkordi E., Owe O., Noll J. Security and Privacy in IoT Systems: A Case Study of Healthcare Products. Proceedings of the 2019 13th International Symposium on Medical Information and Communication Technology (ISMICT).

[25] Chen M., Miao Y., Hao Y., Hwang K. (2017). Narrow Band Internet of Things. IEEE Access.

[26] Mentsiev A.U., Magomaev T.R. (2020). Security Threats of NB-IoT and Countermeasures. IOP Conf. Ser. Mater. Sci. Eng..

[27] Mekki K., Bajic E., Chaxel F., Meyer F. (2019). A Comparative Study of LPWAN Technologies for Large-Scale IoT Deployment. ICT Express.

[28] Popli S., Jha R.K., Jain S. (2019). A Survey on Energy Efficient Narrowband Internet of Things (NBIoT): Architecture, Application and Challenges. IEEE Access.

[29] Sneha, Malik P.K., Bilandi N., Gupta A. (2022). Narrow Band-IoT and Long-Range Technology of IoT Smart Communication: Designs and Challenges. Comput. Ind. Eng..

[30] Migabo E.M., Djouani K.D., Kurien A.M. (2020). The Narrowband Internet of Things (NB-IoT) Resources Management Performance State of Art, Challenges, and Opportunities. IEEE Access.

[31] Raza U., Kulkarni P., Sooriyabandara M. (2017). Medical Internet of Things and Big Data in Healthcare. IEEE Commun. Surv. Tutor..

[32] Dimitrov D.V. (2016). Medical Internet of Things and Big Data in Healthcare. Healthc. Inform. Res..

[33] Sultana N., Huq F., Razzaque M.A., Rahman M.M. (2022). User Utility Maximization in Narrowband Internet of Things for Prioritized Healthcare Applications. Sensors.

[34] Malik H., Alam M.M., Le Moullec Y., Kuusik A. (2018). NarrowBand-IoT Performance Analysis for Healthcare Applications. Procedia Comput. Sci..

[35] Routray S.K., Anand S. Narrowband IoT for Healthcare. Proceedings of the 2017 International Conference on Information Communication and Embedded Systems (ICICES).

[36] Manatarinat W., Poomrittigul S., Tantatsanawong P. Narrowband-Internet of Things (NB-IoT) System for Elderly Healthcare Services. Proceedings of the 2019 5th International Conference on Engineering, Applied Sciences and Technology (ICEAST).

[37] Jin X., Chen S., Zhang L., Wan J., Sun X., Zhang X., Xia Z., Bertino E. (2021). A Pervasive Narrow-Band Internet of Things (NB-IoT) Based Health Monitoring Platform for Ambient Assisted Living. Proceedings of the Advances in Artificial Intelligence and Security.

[38] Almusallam M., Soudani A. (2019). Embedded Solution for Atrial Fibrillation Detection Using Smart Wireless Body Sensors. IEEE Sens. J..

[39] Huda N., Khan S., Abid R., Shuvo S.B., Labib M.M., Hasan T. A Low-Cost, Low-Energy Wearable ECG System with Cloud-Based Arrhythmia Detection. Proceedings of the 2020 IEEE Region 10 Symposium (TENSYMP).

[40] Faust O., Kareem M., Shenfield A., Ali A., Acharya U.R. (2020). Validating the Robustness of an Internet of Things Based Atrial Fibrillation Detection System. Pattern Recognit. Lett..

[41] Shao M., Zhou Z., Bin G., Bai Y., Wu S. (2020). A Wearable Electrocardiogram Telemonitoring System for Atrial Fibrillation Detection. Sensors.

[42] Cañón-Clavijo R.E., Montenegro-Marin C.E., Gaona-Garcia P.A., Ortiz-Guzmán J. (2023). IoT Based System for Heart Monitoring and Arrhythmia Detection Using Machine Learning. J. Healthc. Eng..

[43] Reissenberger P., Serfözö P., Piper D., Juchler N., Glanzmann S., Gram J., Hensler K., Tonidandel H., Börlin E., D’Souza M. (2023). Determine Atrial Fibrillation Burden with a Photoplethysmographic Mobile Sensor: The Atrial Fibrillation Burden Trial: Detection and Quantification of Episodes of Atrial Fibrillation Using a Cloud Analytics Service Connected to a Wearable with Photoplethysmographic Sensor. Eur. Heart J. Digit. Health.

[44] Banerjee P.S., Karmakar A., Dhara M., Ganguly K., Sarkar S. (2021). A Novel Method for Predicting Bradycardia and Atrial Fibrillation Using Fuzzy Logic and Arduino Supported IoT Sensors. Med. Nov. Technol. Devices.

[45] MAX30003WING Evaluation Board|Analog Devices. https://www.analog.com/en/resources/evaluation-hardware-and-software/evaluation-boards-kits/max30003wing.html#eb-overview.

[46] Pan J., Tompkins W.J. (1985). A Real-Time QRS Detection Algorithm. IEEE Trans. Biomed. Eng..

[47] NUCLEO-F401RE-STM32 Nucleo-64 Development Board with STM32F401RE MCU, Supports Arduino and ST Morpho Connectivity-STMicroelectronics. https://www.st.com/en/evaluation-tools/nucleo-f401re.html.

[48] Arm Cortex-M4-Microcontrollers-STMicroelectronics. https://www.st.com/content/st_com/en/arm-32-bit-microcontrollers/arm-cortex-m4.html.

[49] STEVAL-STMODLTE-LTE Connectivity Expansion Board for STMod+ Connector Compatible Evaluation Boards-STMicroelectronics. https://www.st.com/en/evaluation-tools/steval-stmodlte.html.

[50] Sarkar S., Ritscher D., Mehra R. (2008). A Detector for a Chronic Implantable Atrial Tachyarrhythmia Monitor. IEEE Trans. Biomed. Eng..

[51] ThingSpeak Documentation-MathWorks. https://it.mathworks.com/help/thingspeak/index.html?lang=en.

[52] Mainardi L., Sörnmo L., Cerutti S. (2022). Understanding Atrial Fibrillation: The Signal Processing Contribution, Part II.

[53] Babaeizadeh S., Gregg R.E., Helfenbein E.D., Lindauer J.M., Zhou S.H. (2009). Improvements in Atrial Fibrillation Detection for Real-Time Monitoring. J. Electrocardiol..

[54] Lake D.E., Moorman J.R. (2011). Accurate Estimation of Entropy in Very Short Physiological Time Series: The Problem of Atrial Fibrillation Detection in Implanted Ventricular Devices. Am. J. Physiol. Heart Circ. Physiol..

[55] Colloca R. Implementation and Testing of Atrial Fibrillation Detectors for a Mobile Phone Application-Master Thesis. https://www.politesi.polimi.it/handle/10589/78201.

[56] Linker D.T. (2009). Long-Term Monitoring for Detection of Atrial Fibrillation. U.S. Patent.

[57] Goldberger A.L., Amaral L.A., Glass L., Hausdorff J.M., Ivanov P.C., Mark R.G., Mietus J.E., Moody G.B., Peng C.K., Stanley H.E. (2000). PhysioBank, PhysioToolkit, and PhysioNet: Components of a New Research Resource for Complex Physiologic Signals. Circulation.

[58] Dash S., Chon K.H., Lu S., Raeder E.A. (2009). Automatic Real Time Detection of Atrial Fibrillation. Ann. Biomed. Eng..

[59] McGrath M.J., Scanaill C.N., McGrath M.J., Scanaill C.N. (2013). Regulations and Standards: Considerations for Sensor Technologies. Sensor Technologies: Healthcare, Wellness, and Environmental Applications.

[60] Zakerabasali S., Ayyoubzadeh S.M. (2022). Internet of Things and Healthcare System: A Systematic Review of Ethical Issues. Health Sci. Rep..

[61] Centracchio J., Parlato S., Esposito D., Bifulco P., Andreozzi E. (2023). ECG-Free Heartbeat Detection in Seismocardiography Signals via Template Matching. Sensors.

[62] Parlato S., Centracchio J., Esposito D., Bifulco P., Andreozzi E. (2023). ECG-Free Heartbeat Detection in Seismocardiography and Gyrocardiography Signals Provides Acceptable Heart Rate Variability Indices in Healthy and Pathological Subjects. Sensors.

[63] Parlato S., Centracchio J., Esposito D., Bifulco P., Andreozzi E. (2023). Heartbeat Detection in Gyrocardiography Signals without Concurrent ECG Tracings. Sensors.

[64] Andreozzi E., Fratini A., Esposito D., Naik G., Polley C., Gargiulo G.D., Bifulco P. (2020). Forcecardiography: A Novel Technique to Measure Heart Mechanical Vibrations onto the Chest Wall. Sensors.

[65] Andreozzi E., Centracchio J., Punzo V., Esposito D., Polley C., Gargiulo G.D., Bifulco P. (2021). Respiration Monitoring via Forcecardiography Sensors. Sensors.

[66] Centracchio J., Andreozzi E., Esposito D., Gargiulo G.D., Bifulco P. (2022). Detection of Aortic Valve Opening and Estimation of Pre-Ejection Period in Forcecardiography Recordings. Bioengineering.

[67] Andreozzi E., Centracchio J., Esposito D., Bifulco P. (2022). A Comparison of Heart Pulsations Provided by Forcecardiography and Double Integration of Seismocardiogram. Bioengineering.

[68] Centracchio J., Parlato S., Esposito D., Andreozzi E. (2024). Accurate Localization of First and Second Heart Sounds via Template Matching in Forcecardiography Signals. Sensors.

